# Validation of the Arabic Version of the Summary of Diabetes Self-Care Activities Scale Among Type 2 Diabetes Patients in Saudi Arabia

**DOI:** 10.7759/cureus.71813

**Published:** 2024-10-18

**Authors:** Bander Alshehri

**Affiliations:** 1 Medicine, University of Jeddah, Jeddah, SAU

**Keywords:** diabetes, reliability, sdsca instrument, self-management, self-monitoring

## Abstract

Background: The Summary of Diabetes Self-Care Activities (SDSCA) scale is an extensively used instrument for assessing self-care behaviors in diabetic patients. This study aimed to validate the Arabic version of the SDSCA scale in type 2 diabetes to ensure its reliability and applicability in an Arabic-speaking population.

Method: A longitudinal study design was implemented, and initially, 239 participants were selected. The English version of the SDSCA scale was translated by experts using forward and backward translation, and the final version was approved by six expert panels. The final Arabic version was distributed among the type 2 diabetes patients. Psychometric evaluation was performed by the assessment of internal consistency using Cronbach’s alpha, while test-retest reliability was examined using interclass correlation coefficients and exploratory factor analysis.

Results: From 239 participants, 102 were type 2 diabetic patients. Overall, Cronbach’s alpha was 0.72, with the minimum in specific diet (-0.10), while the highest was 0.93 in general diet and blood sugar testing. Meanwhile, moderate to good inter-item and item-to-scale correlations were observed. According to factor analysis, items 1, 2, and 3 loaded heavily on the first dimension; 7 and 8 on the second; 5 and 6 on the third; and 9 and 10 on the fourth item; however, item 4 showed a low factor loading of 0.37 on the third dimension, indicating that this item may not correlate well. The second phase of the factor analysis, except items 3 and 4, exhibits a strong factor structure and captures differences in self-monitoring activity. This strengthens the scale’s validity and reliability as a measurement tool. Overall, test-retest reliability demonstrates the robust reliability of diet (general), exercise, and blood sugar.

Conclusion: The Arabic version of the SDSCA scale is a reliable and valid tool for assessing self-care activities in type 2 diabetes patients in Saudi Arabia, especially in general diet, exercise, and blood sugar testing; however, additional modifications are required to increase the reliability of specific diet domains. This validated scale can be successfully used in clinical and research settings to monitor and enhance the self-care behaviors of Arabic-speaking diabetic patients.

## Introduction

Diabetes is a chronic disease resulting from insufficient insulin production or that cannot be consumed in the body, which results in a long-term metabolic disorder characterized by hyperglycemia [1]. Notably, type 2 diabetes mellitus is a carbohydrate metabolism disorder that occurs due to a major disorder of insulin secretion with or without insulin resistance or relative insulin deficiency and predominant insulin resistance [2]. In 2019, there were 110.1 million people aged 70 years and older living with type 1 and 2 diabetes, and their worldwide prevalence is 23.7% (95% uncertainty interval (UI): 21.8-25.8%), and globally, diabetes caused 181.9 (163-194.7) deaths/100,000 population and 4512.3 (3861.3-5264.2) disability-adjusted life years/100,000 population [3]. Meanwhile, according to 2024 statistics, diabetes affects approximately 537 million adults worldwide between the ages of 20 and 79, accounting for 10.5% of adults in this age group. This number is expected to rise to 643 million by 2030 and 783 million by 2045 worldwide [1]. There are different personal challenges responsible for this upsurge, including (1) lack of resources to afford a healthy lifestyle or food, diabetes monitoring devices, and medication; (2) cultural beliefs regarding the treatment of diabetes; (3) unawareness of difficulty in lifestyle modifications; and (4) psychological issues [4]. Therefore, self-management with lifestyle modification along with medication is very crucial for the achievement of successful diabetes management [5].

Self-management is one of the main and core components of diabetes management; initial improvement can be observed in glucose control by self-management education, self-monitoring, and social support [6]. Evidence also supports self-management effectiveness, particularly during type 2 diabetes for a short time [7]. For this purpose, diabetic patients need to set goals and follow their daily tasks and decisions that are valuable and fit their lifestyle [8]. Socio-environmental conditions related to patient self-management were assessed at the health and community resource level. Support from the healthcare team was measured using 11 items from the patient assessment survey of the chronic care survey [9] and nine items related to chronic healthy eating and exercise, product intake, role resource survey, and seeking community support [10]. Likewise, a 16-item self-care assessment tool for diabetes self-management questionnaires, associated with glycemic control, was developed and validated with good Cronbach’s alpha (0.84) and proved to be a reliable instrument [11]. In addition, the health education impact questionnaire for chronic diseases also has a section of seven items related to self-monitoring and insight, which has been validated and is a reliable tool for assessing chronic conditions, including diabetes [12]. Accurate information on self-care activity levels, as well as glycemic control measures, enables caregivers to monitor patient behavior and adjust interventions to make diabetes management effective; therefore, it is important to develop and implement a validated questionnaire on self-care management among diabetic patients.

The Summary of Diabetes Self-Care Activities (SDSCA) scale is a brief and comprehensive self-reported questionnaire developed for diabetes self-management and includes 10 items related to diet (general and specific), exercise, blood glucose testing, foot care, and smoking [13]. The SDSCA scale has been translated into various languages due to its reliability and effectiveness, for instance, German [14], Chinese [15], Spanish [16], Turkish [17], Urdu [18], and Korean [19], and an Arabic version was validated in Saudi Arabia [20,21]. Furthermore, by providing a culturally appropriate assessment tool, healthcare professionals can better assess and understand their patients’ self-care behaviors, leading to better and more effective interventions. The importance of receiving this tool extends beyond mere academic interest. It has practical implications for clinical practice and public health initiatives aimed at tackling diabetes in the Gulf region.

Therefore, the validation of an Arabic version of the SDSCA scale among type 2 diabetes patients is crucial to ensure culturally and linguistically appropriate assessment tools are available for this population. Accurate and reliable self-care measurement is essential for effective diabetes management and control. By adapting and validating the SDSCA scale for Arabic-speaking patients, healthcare providers can better monitor self-care behaviors, tailor interventions, and improve health outcomes. The study aimed to translate and culturally adapt the SDSCA scale for the assessment of psychometric properties of translated versions among type 2 diabetic patients.

## Materials and methods

Study design and participants

This study adopted a longitudinal design, including type 2 diabetic patients aged over 18 years who had been receiving diabetes treatment for at least one year (Department of Medicine, Jeddah, Saudi Arabia). Additionally, patients were required to be fluent in Arabic. Certain exclusion criteria were also established, such as the presence of severe complications or cognitive impairment.

Questionnaire

The SDSCA questionnaire was designed to assess the frequency of self-monitoring behaviors in the past seven days by individuals with diabetes. The modified questionnaire comprised 11 items to explore special areas of diabetes self-management: diet (food consumption), exercise, blood glucose monitoring, foot monitoring (foot care), and smoking. To make it more practical and easy to use, a brief version of 10 items was adopted in the present study. Each item is indexed by the number of days spent on a particular behavior, yielding a rating that indicates adherence to self-monitoring activities and a high score corresponding to better performance.

Translation and cultural adaptation

The SDSCA scale was translated into Arabic following the World Health Organization guidelines, which were used to translate and adapt instruments [22]. The procedure required forward translation by two bilingual experts, synthesis of the translations, back-translation by two independent bilingual translators [23], and review by an expert committee to ensure cultural and conceptual equivalence. A pre-test was conducted with 20 type 2 diabetes patients to assess the clarity and relevance of the translated items. Lastly, the final version was ready for validation (Appendices section).

Data collection

A trained interviewer approached patients during their regular visits to the institute. The questionnaire was ordered to consenting individuals, and data were collected on age, gender, education, married status, professional activity, insurance status, and diabetes status. One month later, participants were asked to complete the Arabic SDSCA. Participants needed to complete the questionnaire to be included in the final analysis.

Statistical analysis

Sociodemographic variables were described using frequencies and percentages, and the association among variables was explained with Chi-square. The psychometric analysis focused on the first 10 items of the questionnaire. Internal consistency was measured for each domain and all items using Cronbach’s alpha, with values of ≥0.5 considered acceptable and ≥0.7 considered good. Item-to-scale correlations between subscale scores and their constituent items were evaluated using Spearman’s rank correlation (𝑟), with 𝑟 ≥ 0.4 considered acceptable. Inter-item correlations within each subscale were also assessed using Spearman’s rank correlation. Meanwhile, exploratory factor analysis was performed using principal component analysis to extract factors with an eigenvalue criterion of 1. Factor loadings were obtained using the Varimax rotation method. The Kaiser-Meyer-Olkin measure for sampling adequacy and Bartlett’s test for sphericity were reported as well, along with factor loadings for the items. Test-retest reliability was evaluated using the intraclass correlation coefficient (ICC) for individual items and subscales, with ICC values presented along with confidence intervals. All mentioned statistical analyses were performed using SPSS Statistics version 16.0 (SPSS Inc. Released 2007. SPSS for Windows, Version 16.0. Chicago, SPSS Inc.).

Ethical consideration

The Institutional Bioethics Committee of the Scientific and Medical Research of the University of Jeddah approved the study (approval number: HAP-02-J-094), and written consent was acquired from all study participants. The identity of the participants was kept strictly confidential throughout the study.

## Results

Sociodemographic characteristics

The study involved several sociodemographic variables of 239 participants with significant outcomes (p<0.05). The age distribution showed that 53 participants (22.2%) were aged 18-39, 129 (54%) were aged 40-60, 45 (18.8%) were aged 60-70, and 12 (5%) were older than 70. Likewise, 91 (38.1%) of male and 148 (61.9%) of female participants were included in the study. Most participants were primarily married (175, 73.2%), followed by unmarried (30, 12.6%), separated (20, 8.4%), and 14 (5.9%) widowed. Education levels revealed that 52 (21.8%) had postgraduate degrees, 140 (58.6%) had college degrees, 35 (14.6%) had secondary education, and 12 (5%) had no formal education. Meanwhile, employment status indicated that 87 (36.4%) were employed, 58 (24.3%) were unemployed, 41 (17.2%) were in free work, 48 (20.1%) were retired, and five (2.1%) were students. Diabetes status showed 102 (42.7%) with diabetes, 104 (43.5%) without, and 33 (13.8%) uncertain, while smoking status indicated 34 (14.2%) smokers and 68 (28.5%) non-smokers. However, a non-significant difference (p=0.08) was observed in insurance status, as 106 (44.4%) of participants were insured and 133 (55.6%) were uninsured (Table 1).

**Table 1:** Demographic characteristics of study participants (N=239)

Study variables	Frequency	% age	p-value
Age			
18-39	53	22.2	<0.05
40-60	129	54	
60-70	45	18.8	
>70	12	5	
Gender			
Male	91	38.1	<0.05
Female	148	61.9	
Marital status			
Married	175	73.2	<0.05
Separate	20	8.4	
Bachelor	30	12.6	
Widower	14	5.9	
Education level			
Postgraduate	52	21.8	<0.05
College degree	140	58.6	
Secondary	35	14.6	
Without formal education	12	5	
Employment status			
Employee	87	36.4	<0.05
Unemployed	58	24.3	
Free work	41	17.2	
Retired	48	20.1	
Student	5	2.1	
Insurance status			
Yes	106	44.4	0.08
No	133	55.6	
Diabetes status			
Yes	102	42.7	<0.05
No	104	43.5	
Maybe	33	13.8	
Smoking status			
Yes	34	14.2	<0.05
No	68	28.5	

Psychometric properties

Table 2 presents the internal consistency of various subscales from the SDSCA measures. It evaluates the subscales of diet (general and specific), exercise, blood sugar testing, and foot care. For the general diet subscale, two items show a high item-to-scale correlation (1 and 0.86, respectively) and an inter-item correlation of 0.86, resulting in a high Cronbach’s alpha of 0.93, representing excellent internal consistency. In contrast, the specific diet subscale includes two items with lower item-to-scale correlations (0.39 and -0.13), an inter-item correlation of -0.05, and a very low Cronbach’s alpha of -0.10, showing poor consistency. The exercise subscale has two items with moderate item-to-scale correlations (0.33 and 0.29) with a 0.73 inter-item correlation and a Cronbach’s alpha of 0.86, showing good consistency. The blood sugar testing subscale includes two items with item-to-scale correlations of 0.10 and 0.18, an inter-item correlation of 0.85, and a high Cronbach’s alpha of 0.93, indicating excellent consistency. Lastly, the foot-care subscale, with two items showing item-to-scale correlations of 0.25 and 0.27, has an inter-item correlation of 0.58 and a Cronbach’s alpha of 0.69, indicating acceptable consistency. The overall Cronbach’s alpha for all items combined is 0.72, reflecting satisfactory internal consistency across the entire measure.

**Table 2:** Internal consistency of the SDSCA measure subscales (N=102) * non-significant, ** significant at 0.01 SDSCA: Summary of Diabetes Self-Care Activities

Scales	Items	Item to scale correlation	Inter-item correlation	Cronbach’s alpha
Diet				
General	1	1	0.86	0.93
	2	0.86^**^		
Specific	3	0.39^**^	-0.05^*^	-0.10
	4	-0.13		
Exercise	5	0.33^**^	0.73	0.86
	6	0.29^**^		
Blood sugar testing	7	0.10	0.85	0.93
	8	0.18		
Foot care	9	0.25	0.58	0.69
	10	0.27^**^		
All items				0.72

Factor analysis was done in two steps. Regarding the first step, the 10 items were involved in principal factor analysis. Kaiser-Meyer-Olkin’s measure for sampling adequacy was 0.59, and Bartlett’s test of sphericity was statistically significant (p<0.01). Items 1, 2, and 3 load highly on the first component, with factor loadings of 0.89, 0.91, and 0.60, respectively. Items 7 and 8 load highly on the second component, each with a factor loading of 0.92. Items 5 and 6 load on the third component with factor loadings of 0.82 and 0.88, while items 9 and 10 load on the fourth component with a factor loading of 0.85. Item 4 has a low loading of 0.37 on the third component. The eigenvalues for the components are 3.15, 1.90, 1.37, and 1.15, respectively, explaining 31.53%, 19.08%, 13.76%, and 11.59% of the variance. The cumulative variance described by these components is 75.97% (Table 3).

**Table 3:** Outcomes of the exploratory factor analysis of the Moroccan SDSCA items (10-item version): factor loadings and explained variance (N=102) SDSCA: Summary of Diabetes Self-Care Activities

Items	Components			
	1	2	3	4
1	0.89	-	-	-
2	0.91	-	-	-
3	0.60	-	-	-
4	-	-	0.37	-
5	-	-	0.82	-
6	-	-	0.88	-
7	-	0.92	-	-
8	-	0.92	-	-
9	-	-	-	0.85
10	-	-	-	0.85
Eigenvalues	3.15	1.90	1.37	1.15
% of variance	31.53	19.08	13.76	11.59
Cumulative % of variance	31.53	50.62	64.38	75.97

Regarding the second step, factor analysis was performed after deleting items 3 and 4 (specific diet subscale) due to their weak performance in the first step and their weak internal consistency. Kaiser-Meyer-Olkin’s measure for sampling adequacy was 0.57, and Bartlett’s test of sphericity was significant (p<0.01). The analysis identified four components, with items 1, 2, 7, and 8 loading heavily on component 1 (factor loadings of 0.94, 0.95, 0.96, and 0.95, respectively). Items 5 and 6 loaded strongly on component 2 (0.90 and 0.92). Items 9 and 10 loaded on component 3 (0.88 and 0.84). No items loaded significantly on component 4. The eigenvalues for the components were 2.85, 1.89, 1.31, and 1.07, explaining 35.62%, 23.62%, 16.44%, and 13.40% of the variance, respectively, with a cumulative variance of 89.10% (Table 4).

**Table 4:** Outcomes of the exploratory factor analysis of the Moroccan SDSCA items (eight-item version: items 3 and 4 deleted): factor loadings and explained variance (N=102) SDSCA: Summary of Diabetes Self-Care Activities

Items	Components			
	1	2	3	4
1	-	0.94	-	-
2	-	0.95	-	-
5	-	-	0.90	-
6	-	-	0.92	-
7	0.96	-	-	-
8	0.95	-	-	-
9	-	-	-	0.88
10	-	-	-	0.84
Eigenvalues	2.85	1.89	1.31	1.07
% of variance	35.62	23.62	16.44	13.40
Cumulative % of variance	35.62	59.25	75.69	89.10

Table 5 presents the test-retest reliability assessed using ICCs and their 95% confidence intervals (CI). The general diet domain showed an overall ICC of 0.90, with item 1 at 0.87 (CI: 0.82-0.91) and item 2 at 0.93 (CI: 0.90-0.95). The specific diet domain had a low overall ICC of -0.35, with item 3 at -0.50 (CI: -0.24-0.14) and item 4 at -0.10 (CI: -0.63-0.25). The exercise domain showed an ICC of 0.81, with item 5 at 0.76 (CI: 0.66-0.83) and item 6 at 0.86 (CI: 0.79-0.90). The blood sugar testing domain had a high ICC of 0.90, with item 7 at 0.87 (CI: 0.81-0.91) and item 8 at 0.93 (CI: 0.90-0.95). The foot care domain showed a moderate ICC of 0.61, with item 9 at 0.53 (CI: 0.38-0.66) and item 10 at 0.69 (CI: 0.55-0.79).

**Table 5:** Test-retest reliability of the Moroccan SDSCA items (N=102) SDSCA: Summary of Diabetes Self-Care Activities, ICC: intraclass correlation, CI: confidence interval

Domains	Items	ICC	95% CI
General diet		0.90	
	1	0.87	0.82-0.91
	2	0.93	0.90-0.95
Specific diet		-0.35	
	3	-0.50	-0.24-0.14
	4	-0.10	-0.63-0.25
Exercise		0.81	
	5	0.76	0.66-0.83
	6	0.86	0.79-0.90
Blood sugar testing		0.90	
	7	0.87	0.81-0.91
	8	0.93	0.90-0.95
Foot care		0.61	
	9	0.53	0.38-0.66
	10	0.69	0.55-0.79

## Discussion

The validation of the Arabic version of the SDSCA scale among type 2 diabetes patients provides critical insights into the applicability and reliability of this tool in Arabic-speaking populations. Our findings indicate that the translated scale maintains good overall internal consistency and construct validity with 0.72 Cronbach’s alpha, making it a valuable instrument for assessing self-care activities in this demographic. However, items 3 and 4 had very low Cronbach’s alpha (-0.10). Our findings are well supported by the findings of another study, which observed low internal consistency among items 3 and 4 (Cronbach’s alpha=0.16) [24]. Similarly, low internal consistency with 0.40 Cronbach’s alpha was observed in the same items related to specific diets [13]. Meanwhile, Urdu-translated SDSCA also had a high overall internal consistency with a Cronbach’s alpha of 0.79 [18]. Furthermore, the Spanish version also had an overall internal consistency with a Cronbach’s alpha of 0.68 [16]. Moreover, a moderate overall internal consistency was observed in Korean-translated SDSCA with a Cronbach’s alpha of 0.69 [19]. The low reliability of these items may be attributed to several factors, including cultural differences in terms of food consumption in the understanding and practice of specific self-care activities or the variability in how these activities are performed among individuals. Additionally, these items may not align well with the overall construct of the scale or may be influenced by external factors not accounted for in the questionnaire, leading to inconsistent responses. In addition, some of the studies excluded specific diets (items 3 and 4), and it may be due to eating habits, as the local population likes to consume high-fatty stuff in a daily routine, which may give unreliable consistency and outcomes [13,18]. Additionally, other studies also faced the same problem with items 3 and 4 (specific diet) [16,19].

The factor analysis of the 10 items in the SDSCA scale revealed four distinct components, indicating a multifactorial structure. Items 1, 2, and 3 loaded highly on the first component, items 7 and 8 on the second, items 5 and 6 on the third, and items 9 and 10 on the fourth component. Item 4 showed a low factor loading of 0.37 on the third component, suggesting that it may not align well with this factor. These results support the scale’s construct validity but highlight the need for potential refinement of item 4 to enhance the overall fit and reliability of the scale in the first step. A similar challenge was encountered with item 4 in the Malay version of the questionnaire designed for children and adolescents. In their factor analysis, item 4 loaded on the “blood sugar checking” subscale, as it did at the beginning of our factor analysis. In the final Malay version, item 4 was replaced by another item from the extended version [25]. Meanwhile, the second step of factor analysis in the present study indicates that the revised scales, except items 3 and 4, exhibit a robust factor structure and capture a substantial proportion of the variance in self-care activities among type 2 diabetics, thus providing its validity and reliability as a measurement tool is high.

In the present study, the test-retest reliability assessment demonstrates varying degrees of consistency across different domains. Overall, it highlights the robust reliability of the general diet, exercise, and blood sugar testing domains while indicating a need for further refinement in the specific diet and foot care domains to enhance their reliability. Likely, maybe these activities are more consistently practiced and understood among patients. High ICC values in these domains suggest that participants’ self-reported behaviors were stable over time, reflecting consistent adherence to these self-care practices. Similarly, during the validation of the Spanish version of SDSCA, a good correlation was observed [16]. Furthermore, another study also observed a good and positive correlation and agreed with our findings [19]. Moreover, a significant test-retest reliability of 0.91 was observed during the validation of the Urdu-translated SDSCA version [18].

This validation is particularly important given the growing prevalence of diabetes in Arabic-speaking regions and the need for culturally appropriate tools to enhance diabetes management and patient education. However, items related to specific diets (items 3 and 4) need to be excluded as they proved challenging for participants to understand.

The current study has several limitations, such as the study sample may not be fully representative of the broader Arabic-speaking population with type 2 diabetes, which limits the generalizability of the outcomes. Another limitation is the reliance on self-reported data. This may introduce bias or inaccuracy due to recall issues or social desirability. Finally, the study may not take into account regional differences within Saudi Arabia. This may affect self-care behavior and the applicability of the scale to different patient subgroups.

## Conclusions

The validation of the Arabic version of the SDSCA scale in type 2 diabetic patients showed that this instrument is generally reliable and suitable for screening self-care activities in the study population. The study showed good overall internal consistency with a Cronbach alpha of 0.72. General diet, exercise, and blood sugar monitoring components showed robust internal reliability in the test-retest, but specific aspects of diet showed low reliability, suggesting that further modifications are needed to increase their consistency. These findings highlight the importance of culturally adapted instruments for the effective assessment of diabetes care and support the use of the Arabic SDSCA scale in clinical and research settings to improve diabetes management in Arabic-speaking patients in Saudi Arabia.

## Appendices

**Table 6:** Final version of the SDSCA questionnaire SDSCA: Summary of Diabetes Self-Care Activities

Items	English	Arabic
	Diet	
1	In the previous seven days, how many days did you follow a healthy eating plan?	في السبعة ايام السابقة , ماهو عدد الايام الذي قمت فيه بتناول 5 حصص او اكثر من الخضروات و الفاكهة ؟
2	On average, how many days a week did you follow your eating plan during the past month?	في السبعة ايام السابقة , ماهو عدد الايام الذي قمت فيه بتناول اغذية عالية الدسم مثل اللحوم الحمراء و منتجات الالبان كاملة الدسم؟
3	In the past seven days, how many days did you eat 5 or more servings of vegetables and fruits?	في السبعة ايام السابقة , كم عدد الايام التي مارست فيها انشطة بدنية لمدة 30 دقيقة علي الاقل ؟
4	In the previous seven days, how many days did you eat high-fat foods such as red meat and full-fat dairy products?	(متضمن السباحة او المشي او ركوب الدراجة) غيرالانشطة المعتادة الذي تقوم بها في منزلك او في عملك ؟
	Exercise	
5	In the past seven days, how many days did you engage in physical activity for at least 30 minutes?	في السبعة ايام السابقة , كم عدد الايام التي فحصت فيها السكر بالدم ؟
6	In the previous seven days, how many days did you do a specific exercise?	في السبعة ايام السابقة , كم عدد الايام التي فحصت فيها السكر بالدم حسب العدد الذي اوصي به طبيبك ؟
	Blood sugar testing	
7	In the previous seven days, how many days did you check your blood sugar?	في السبعة ايام السابقة , كم عدد الايام التي فحصت فيها قدميك ؟
8	In the previous seven days, how many days did you check your blood sugar, according to the number recommended by your doctor?	في السبعة ايام السابقة , كم عدد الايام التي فحصت فيها حذائك من الداخل
	Foot care	
9	In the past seven days, how many days did you examine your feet?	هل قمت بالتدخين و لو حتي لنفس واحد في السبعة ايام السابقة ؟
10	In the previous seven days, how many days did you check the inside of your shoes?	إذا كانت الإجابة نعم , ما هو متوسط عدد السجائرالتي تدخنها في اليوم

## References

[1] Kumar A, Gangwar R, Zargar AA, Kumar R, Sharma A (2024). Prevalence of diabetes in India: a review of IDF diabetes atlas 10th edition. Curr Diabetes Rev.

[2] Ergasheva Gulshan T (2024). Risk factors for developing Type 2 diabetes mellitus.. ОБРАЗОВАНИЕ НАУКА И ИННОВАЦИОННЫЕ ИДЕИ В МИРЕ.

[3] Jiang S, Yu T, Di D, Wang Y, Li W (2024). Worldwide burden and trends of diabetes among people aged 70 years and older, 1990-2019: a systematic analysis for the Global Burden of Disease Study 2019. Diabetes Metab Res Rev.

[4] Whittemore R, Vilar-Compte M, De La Cerda S (2019). Challenges to diabetes self-management for adults with type 2 diabetes in low-resource settings in Mexico City: a qualitative descriptive study. Int J Equity Health.

[5] Borse SP, Chhipa AS, Sharma V, Singh DP, Nivsarkar M (2021). Management of type 2 diabetes: current strategies, unfocussed aspects, challenges, and alternatives. Med Princ Pract.

[6] Kisokanth G, Prathapan S, Indrakumar J, Joseph J (2013). Factors influencing self-management of diabetes mellitus; a review article. J Diabetol.

[7] Norris SL, Engelgau MM, Narayan KM (2001). Effectiveness of self-management training in type 2 diabetes: a systematic review of randomized controlled trials. Diabetes Care.

[8] Funnell MM, Anderson RM (2004). Empowerment and self-management of diabetes. Clin Diabetes.

[9] Glasgow RE, Wagner EH, Schaefer J, Mahoney LD, Reid RJ, Greene SM (2005). Development and validation of the patient assessment of chronic illness care (PACIC). Med Care.

[10] Glasgow RE, Strycker LA, Toobert DJ, Eakin E (2000). A social-ecologic approach to assessing support for disease self-management: the chronic illness resources survey. J Behav Med.

[11] Schmitt A, Gahr A, Hermanns N, Kulzer B, Huber J, Haak T (2013). The diabetes self-management questionnaire (DSMQ): development and evaluation of an instrument to assess diabetes self-care activities associated with glycaemic control. Health Qual Life Outcomes.

[12] Osborne RH, Elsworth GR, Whitfield K (2007). The health education impact questionnaire (heiQ): an outcomes and evaluation measure for patient education and self-management interventions for people with chronic conditions. Patient Educ Couns.

[13] Toobert DJ, Hampson SE, Glasgow RE (2000). The summary of diabetes self-care activities measure: results from 7 studies and a revised scale. Diabetes Care.

[14] Kamradt M, Bozorgmehr K, Krisam J (2014). Assessing self-management in patients with diabetes mellitus type 2 in Germany: validation of a German version of the summary of diabetes self-care activities measure (SDSCA-G). Health Qual Life Outcomes.

[15] Tang J, Wu T, Hu X, Gao L (2021). Self-care activities among patients with type 2 diabetes mellitus: a cross-sectional study. Int J Nurs Pract.

[16] Vincent D, McEwen MM, Pasvogel A (2008). The validity and reliability of a Spanish version of the summary of diabetes self-care activities questionnaire. Nurs Res.

[17] Peker A, Mert S, Demirhan Y (2022). Diabetes Health promotion self-care scale: reliability and validity of the Turkish version. Osmangazi Tıp Dergisi.

[18] Ansari RM, Harris MF, Hosseinzadeh H, Zwar N (2020). The summary of an Urdu version of diabetes self-care activities measure: psychometric evaluation and validation. J Prim Care Community Health.

[19] Choi EJ, Nam M, Kim SH, Park CG, Toobert DJ, Yoo JS, Chu SH (2011). Psychometric properties of a Korean version of the summary of diabetes self-care activities measure. Int J Nurs Stud.

[20] Sukkarieh-Haraty O, Howard E (2016). Psychometric properties of the Arabic version of the summary of diabetes self-care activities instrument. Res Theory Nurs Pract.

[21] AlJohani KA, Kendall GE, Snider PD (2016). Psychometric evaluation of the summary of diabetes self-care activities-Arabic (SDSCA-Arabic): translation and analysis process. J Transcult Nurs.

[22] Nikpajouh A, Shahrbaf MA, Doayie M (2018). Health promoting hospitals in Iran: Persian translation, cultural adaptation, content and face validation of selfassessment form of the standards of health promoting hospitals affiliated to the World Health Organization. Med J Islam Repub Iran.

[23] Beaton DE, Bombardier C, Guillemin F, Ferraz MB (2000). Guidelines for the process of cross-cultural adaptation of self-report measures. Spine.

[24] Adarmouch L, Sebbani M, Elyacoubi A, Amine M (2016). Psychometric properties of a Moroccan version of the summary of diabetes self-care activities measure. J Diabetes Res.

[25] Jalaludin M, Fuziah M, Hong J, Mohamad Adam B, Jamaiyah H (2012). Reliability and validity of the revised summary of diabetes self-care activities (SDSCA) for Malaysian children and adolescents. Malays Fam Physician.

